# Highly-Bioreactive Silica-Based Mesoporous Bioactive Glasses Enriched with Gallium(III)

**DOI:** 10.3390/ma11030367

**Published:** 2018-03-02

**Authors:** Sandra Sanchez-Salcedo, Gianluca Malavasi, Antonio J. Salinas, Gigliola Lusvardi, Luca Rigamonti, Ledi Menabue, Maria Vallet-Regi

**Affiliations:** 1 Dpto. Química en Ciencias Farmacéuticas, Instituto de Investigación Sanitaria Hospital 12 de Octubre, Universidad Complutense de Madrid, 28040 Madrid, Spain; sansanch@ucm.es (S.S.-S.); vallet@ucm.es (M.V.-R.); 2 Centro Investigación Biomédica en Red Bioingeniería, Biomateriales y Nanomedicina (CIBER-BBN), 28040 Madrid, Spain; 3 Department of Chemical and Geological Sciences, University of Modena and Reggio Emilia, Via G. Campi 103, 41125 Modena, Italy; gigliola.lusvardi@unimore.it (G.L.); luca.rigamonti@unimore.it (L.R.); ledi.menabue@unimore.it (L.M.)

**Keywords:** gallium ions, mesoporous glasses, glass structure, in vitro bioactivity, antibacterial capability

## Abstract

Beneficial effects in bone cell growth and antibacterial action are currently attributed to Ga^3+^ ions. Thus, they can be used to upgrade mesoporous bioactive glasses (MBGs), investigated for tissue engineering, whenever they released therapeutic amounts of gallium ions to the surrounding medium. Three gallium-enriched MBGs with composition (in mol %) *x*SiO_2_–*y*CaO–*z*P_2_O_5_–5Ga_2_O_3_, being *x* = 70, *y* = 15, *z* = 10 for **Ga_1**; *x* = 80, *y* = 12, *z* = 3 for **Ga_2**; and *x* = 80, *y* = 15, *z* = 0 for **Ga_3**, were investigated and compared with the gallium-free 80SiO_2_–15CaO–5P_2_O_5_ MBG (**B**). ^29^Si and ^31^P MAS NMR analyses indicated that Ga^3+^ acts as network modifier in the glass regions with higher polymerization degree and as network former in the zones with high concentration of classical modifiers (Ca^2+^ ions). **Ga_1** and **Ga_2** exhibited a quick in vitro bioactive response because they were coated by an apatite-like layer after 1 and 3 days in simulated body fluid. Although we have not conducted biological tests in this paper (cells or bacteria), **Ga_1** released high but non-cytotoxic amounts of Ga^3+^ ions in Todd Hewitt Broth culture medium that were 140 times higher than the IC90 of *Pseudomonas aeruginosa* bacteria, demonstrating its potential for tissue engineering applications.

## 1. Introduction

The inclusion of so-called therapeutic ions in silica-based mesoporous bioactive glasses (MBGs) is a subject of current interest because of the beneficial effects they can produce once implanted [1]. The advantages given by these ions are connected to those of the traditional components of bioactive glasses: (i) stimulate the expression of genes of osteoblastic cells [2], (ii) stimulate angiogenesis [3], (iii) join certain antibiotics to produce antimicrobial behaviour [4], and (iv) show anti-inflammatory effects [5]. In this context, Ga^3+^ ions are being investigated to be included in MBGs to obtain 3D scaffolds for bone regeneration—in particular for the defects treatment due to their propitious effects in bone cell growth and thanks to their antibacterial action. In fact, bone can be considered as a target organ for gallium, as this metal can be found at sites of rapid bone remodelling, such as active metaphyseal growth plate and healing fractures [6,7]. In 2009, Ma et al. reported that gallium improves bone strength and calcium content in osteopenic rats, probably by decreasing the rate of bone resorption [8]. Indeed, gallium was already approved by the FDA for the treatment of hypercalcemia and also used for treating Paget’s bone disease [9,10]. More recently, gallium has emerged as a new-generation antibacterial ion that may be useful in treating and preventing localized infections. In fact, when Ga^3+^ is exogenously supplied to bacterial cells, it can replace Fe^3+^, perturbing the bacterial metabolism. Therefore, the interest in Ga^3+^-based antibacterial materials is a complementary strategy for the development of novel drugs to tackle multidrug-resistant bacteria [11,12,13,14,15]. Moreover, in contrast with silver and copper, gallium can be metabolically active by the substitution of iron in many biological systems, due to the chemical similarities of Ga^3+^ with Fe^3+^ in terms of charge, ionic radius, and electronic configuration [12]. As a result, Ga^3+^ exhibits these positive consequences without inducing cytotoxicity [16].

In MBGs’ structure, Ga^3+^ ions exhibit an intermediate behaviour between network formers and network modifiers. In a previous paper, MBGs doped with up to 3.5 mol % of Ga_2_O_3_ were synthesized and characterized [17]. However, these glasses only released imperceptible amounts of Ga^3+^ ions in the medium (below 0.05 ppm in simulated body fluid, SBF), hampering the possible bactericidal action of these materials. That study [17] revealed that the release of Ga^3+^ from the final MBG does not depend on the amount added to the glass composition, but on its location in the glass network, which favours or does not favour the liberation in the surrounding medium. Nevertheless, there are several reported examples of gallium-doped glasses, also with mesoporous structure [18] and with similar composition to the more soluble phosphate glasses obtained by melting [19], where the high amount of released gallium ions in the physiological medium produced antibacterial effects, even above the biocompatible limit. Considering the adequate bioactivity and biocompatibility of MBGs, as well as their high specific surface area which enables the adsorption of therapeutic molecules and their subsequent controlled release [12,13,14], the possibility of releasing the right amount of gallium to inhibit biofilm formation on scaffolds for bone tissue defects is of great interest.

All of these results have highlighted the importance of studying the different positions that can be occupied by gallium ions in a network of mesoporous glass. Thus, in this paper, three mesoporous glasses in the quaternary system *x*SiO_2_–*y*CaO–*z*P_2_O_5_–5Ga_2_O_3_ were synthesized, and their chemical structure was investigated by NMR spectroscopy. In particular, the three glasses have the following composition (in mol %): *x* = 70, *y* = 15, *z* = 10 for **Ga_1**; *x* = 80, *y* = 12, *z* = 3 for **Ga_2**; and *x* = 80, *y* = 15, *z* = 0 for **Ga_3**, thus containing fixed 5 mol % of Ga_2_O_3_ and different P_2_O_5_ contents, 10, 3, and 0 mol %, respectively. These glass compositions were selected in the search for glasses able to exhibit different Ga^3+^ release patterns, and they were compared with gallium-free 80SiO_2_–15CaO–5P_2_O_5_ MBG (**B**). The main objective of this study is the correlation of the location of the gallium ions in the glass network with their bioactive behaviour, and the amount of Ga^3+^ ions released after soaking in common cell culture media, such as Dulbecco’s Modified Eagle Medium (DMEM) and Todd Hewitt Broth (THB). Although we did not conduct biological tests (cells or bacteria), the final goal of this study is to check if these glasses—which maintain a mesoporous structure—are able to exhibit in vitro bioactivity as well as release gallium ions at the bactericidal-activity levels while still remaining under the cytotoxic level.

## 2. Results

### 2.1. Synthesis of MBGs and Their Characterization

The MBGs studied in this paper were obtained by evaporation-induced self-assembly (EISA) process according to a previously-described method [17], and their compositions are reported in Table 1. The gallium-containing glass compositions were proposed in order to increase the Ga^3+^ ions released during in vitro biocompatibility and antibacterial assays. In particular, in all samples the Ga_2_O_3_ concentration was raised up to 5 mol % with respect to the amount of 3.5 mol % in the previously reported glass [17]. In **Ga_1**, the silica content was reduced while increasing P_2_O_5_ in order to decrease the polymerization degree of the glass network; in fact, it is well-known that P_2_O_5_ is mainly present as an orthophosphate unit (Q^0^ species) [20]. In **Ga_2** and **Ga_3**, we had to progressively reduce the P_2_O_5_ content, since it was demonstrated that calcium can play a different role depending on the bioactive glass composition [21]: in fact, in the ternary SiO_2_–CaO–P_2_O_5_ system, Ca^2+^ ions tend to cluster around [PO_4_] orthophosphate units, while Ca^2+^ ions in binary SiO_2_–CaO glass break the silica network. These two different structural roles of calcium ions in the ternary and binary glasses cause a lower amount of released Ca^2+^ ions during bioactivity tests in the first system with respect to the second. In this scenario, Ga^3+^ ions could play a similar role as Ca^2+^ ions, and the lower amount or absence of P_2_O_5_ in the glass composition could favour the release of Ga^3+^ ions associated to the silica network.

**Figure 1:**
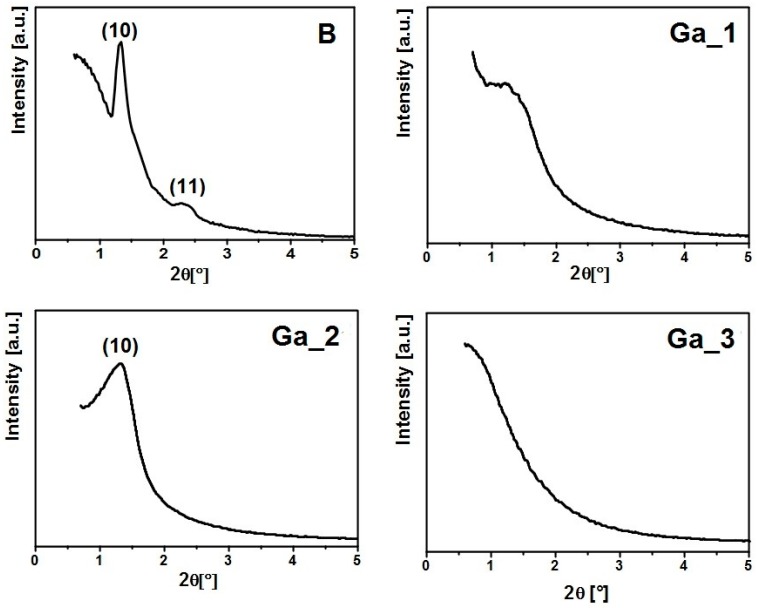
Low-angle powder X-ray diffraction (PXRD) patterns of **B**, **Ga_1**, **Ga_2**, and **Ga_3**.

Figure 1 reports low-angle powder X-ray diffraction (PXRD) spectra, which are necessary to verify the possible mesoporous order in **B**, **Ga_1**, **Ga_2**, and **Ga_3** after calcination at 700 °C. We can observe that **B** produces a sharp diffraction maximum at 2*θ* in the region of 1.3–1.4°, assigned to the (10) reflection, along with a less-resolved peak at about 2.3° that can be ascribed to the (11) reflection as previously reported [22]. The intensity of the PXRD maxima decreases on passing from **B** to **Ga_2**, indicative of a partial deterioration of the mesoporous order [17]. The **Ga_1** shows a poorly resolved peak in the 1.2–1.4° region, hinting at a weaker mesoporous order, while **Ga_3** does not present any diffraction maximum at low angles.

**Figure 2:**
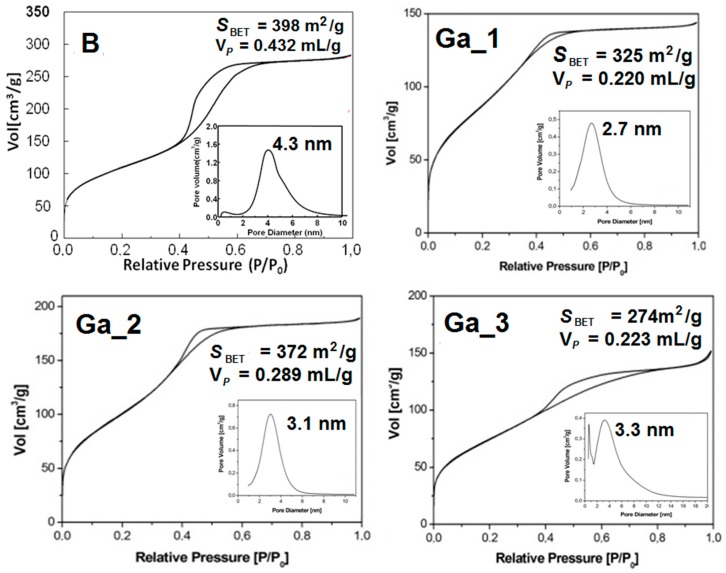
Nitrogen adsorption–desorption isotherms of **B**, **Ga_1**, **Ga_2**, and **Ga_3** with reported *S*_BET_ and *V*_P_ values. Insets: pore diameter distribution curves.

Figure 2 shows the N_2_ adsorption–desorption isotherms and the pore size distribution for **B**, **Ga_1**, **Ga_2**, and **Ga_3**. The reported isotherms can be classified as type IV, which are characteristic of mesoporous material, and the hysteresis loops are of type H in the mesoporous range, which are distinctive of cylindrical pores. Samples **Ga_1** and **Ga_2** display a single-model size distribution centred at 2.7 and 3.1 nm, respectively, while **Ga_3** shows two maxima centred at around 3.3 nm and below 1 nm, in which the latter is probably due to microporosity. The pore size distribution is relatively wide, characteristic of mesoporous glasses obtained by EISA method [23]. The textural properties of **Ga_1**, **Ga_2**, and **Ga_3** (e.g., specific surface area, *S*_BET_, pore diameter, *D*_P_, and total pore volume, *V*_P_) are similar to those of gallium-containing mesoporous glasses up to 3.5 mol % of Ga_2_O_3_ previously reported [17].

**Figure 3:**
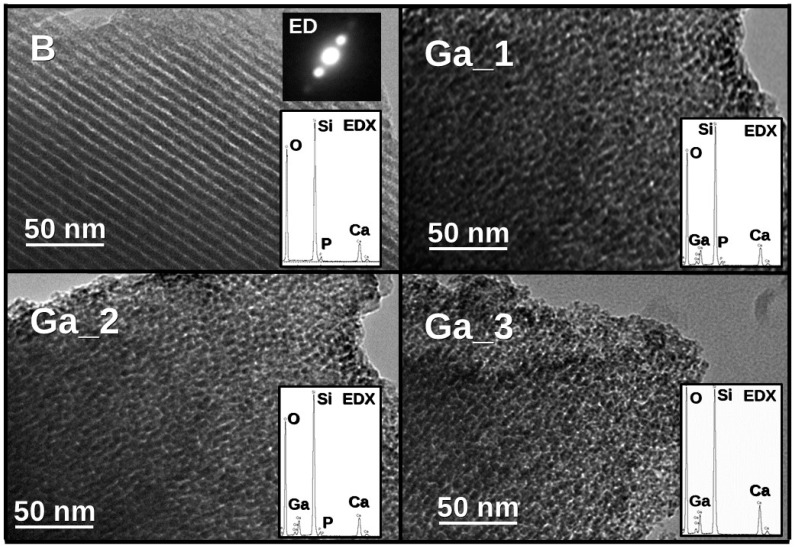
TEM images of **B**, **Ga_1**, **Ga_2**, and **Ga_3** with the corresponding EDX spectra included in the bottom-right corner of the micrographs. The electron diffraction pattern of **B**—the highest ordered sample—is also included in the upper-right corner of the micrograph.

Transmission electron microscopy (TEM) images of **B**, **Ga_1**, **Ga_2**, and **Ga_3** are reported in Figure 3. A typical two-dimensional (2D) hexagonal order mesoporous arrangement channels was shown only by **B**, and this is confirmed by the electronic diffraction shown in the inset. However, also in the images of **Ga_2** it is possible to observe some zones exhibiting mesoporous order. For **Ga_1** and **Ga_3**, a poorly-ordered structure can instead be recognized. Energy-dispersive X-ray (EDX) analysis was also used to determine the experimental composition reported in Table 1 in comparison with the theoretical molar percentages, showing a good resemblance.

^29^Si and ^31^P solid-state magic angle spinning (MAS) nuclear magnetic resonance (NMR) measurements were carried out to further investigate the environments of the network-forming species on these samples at the atomic level. Table 2 and Table 3 show the chemical shifts, deconvoluted peak areas, and silica or phosphorous network connectivity (NC) <Q^n^> for each glass composition. In particular, in Table 2 Q^2^, Q^3^, and Q^4^ represent the silicon atoms (denoted Si*) in (NBO)_2_–Si*–(OSi)_2_, (NBO)–Si*–(OSi)_3_, and Si*(OSi)_4_ (NBO = non-bonding oxygen adjacent to another Si atom), respectively, while in Table 3 Q^0^ and Q^1^ represent phosphorus atoms (denoted P*) in the PO_4_ units of P*–(NBO)_4_ and (NBO)_3_–P*–(OP), respectively.

In the single-pulse ^29^Si MAS NMR spectra, the signals in the region between −111 and −112 ppm come from Q^4^, those from −102 to −103 ppm are given by Q^3^, and those from −92 to −89 ppm by Q^2^. Single-pulse ^29^Si MAS NMR spectroscopy was used to evaluate the NC of mesoporous glasses as a function of the chemical composition. Sample **B** was characterized by a high percentage of Q^4^ and Q^3^ species with NC of 3.52, very similar to that found in iso-compositional mesoporous glasses [22]. The introduction of 5 mol % Ga_2_O_3_ in **Ga_1**, **Ga_2**, and **Ga_3** caused a decrease of network connectivity due to the decrease of the percentage of Q^4^ and the increase in the amount of Q^3^ species. The NC [24] can be also computed using the simplified model proposed by Martin et al. [25]; using this model, the NC value for **B** is 4, assuming that all P is present as orthophosphate units (Q^0^). This value is greater than determined by the <Q^n^> obtained from single-pulse ^29^Si MAS NMR analysis. This is most likely due to the fact that a fraction of P becomes part of the glass network forming Si–O–P bridges. The model proposed in the aforementioned article can be used to calculate NC for glasses that also contain other forming ions in addition to Si, such as Ga. In fact, if we computed the NC values assuming Si and Ga as network former ions, the results obtained were 4.5, 4.04, and 3.78 for **Ga_1**, **Ga_2**, and **Ga_3**, respectively. These values are always greater than found by analysis of the <Q^n^>, but this can be easily explained by the high amount of OH groups present in the glass network. These groups reduce the connectivity. Moreover, another explanation can be made assuming that not all Ga^3+^ ions play the role of network former.

A similar trend was observed in the cross-polarized ^29^Si MAS NMR spectra; however, the network connectivity was lower with respect to that found using the single-pulse analysis. The <Q^n^> determined by cross-polarized MAS NMR spectra was lower than in single-pulse experiments, because the first is mainly sensible to the glass surface rich in H nuclei [26].

The single-pulse ^31^P MAS NMR spectra of the materials show a mean maximum in the range of 1–2 ppm, assigned to the Q^0^ environment typical of an amorphous orthophosphate. A second weak signal located between −5 and −6 ppm falls in the range of Q^1^ tetrahedra, and can be attributed to P–O–Si environments as reported by previous studies [27,28].

### 2.2. In Vitro Bioactivity Assay

**Figure 4:**
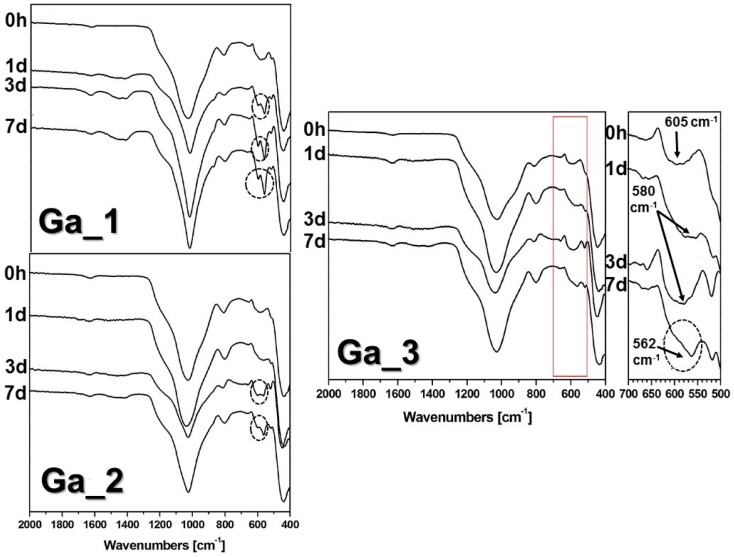
Fourier transform infrared (FTIR) spectra of **Ga_1**, **Ga_2**, and **Ga_3** before (0 h) and after (1 day, 3 days, and 7 days) soaking in simulated body fluid (SBF). Circles show the formation of P–O doublet assigned to crystalline calcium phosphate.

Figure 4 shows the Fourier transform infrared (FTIR) spectra of **Ga_1**, **Ga_2**, and **Ga_3** before and after being soaked in SBF at 37 °C for 1, 3, and 7 days [29]. The FTIR spectra of all glasses before soaking in SBF show intense absorption bands at 1040 and 470 cm^−1^ that correspond to the asymmetric bending vibrations of the Si–O–Si bond, and a band at 800 cm^−1^ attributable to symmetric stretching of the Si–O bond [30]. The weak band at 585 cm^−1^ present in **Ga_1** and **Ga_2** was ascribed to an phosphate group in an amorphous environment, while **Ga_3** presented a band at 605 cm^−1^, attributed to the Ga–O in tetrahedral coordination [31].

Different types of in vitro responses can be recognized for the different samples. In particular, a high in vitro bioactivity was shown by **Ga_1** comparable to that found for **B** [17] (not reported in the present study for the sake of brevity). In fact, already after 1 day of soaking in SBF it was possible to observe the formation of the characteristic doublet at 564 and 602 cm^−1^, usually assigned to crystalline calcium phosphate [32]. **Ga_2** showed an intermediate behaviour, since the doublet at 564 and 603 cm^−1^ appeared after 7 days of soaking in SBF (see Figure 4), while lower in vitro bioactivity was given by **Ga_3** because after 1 day of soaking the formation of a broad peak centred at 580 cm^−1^ (inset in Figure 4) due to the formation of amorphous Ca–P layer could be observed. At longer times, no peak attributed to the crystallization of calcium phosphate appeared, and only a shoulder at 562 cm^−1^ became visible.

**Figure 5:**
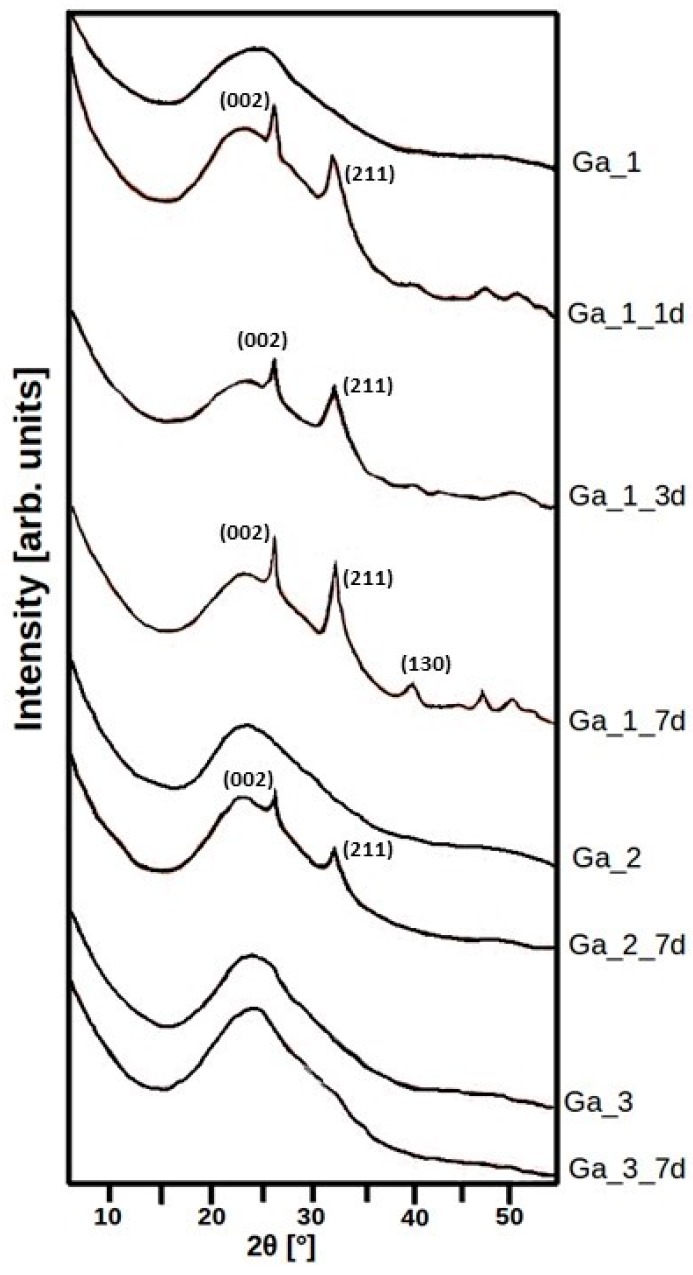
PXRD patterns of **Ga_1**, **Ga_2**, and **Ga_3** after different amounts of time soaking in SBF.

The bioactivity in terms of apatite-like layer (e.g., hydroxycarbonate apatite, HCA) formation after different amounts of time soaking in SBF is reported as PXRD spectra in Figure 5. The peaks at about 32° and 26° in 2*θ* are attributed to the (211) and (002) reflections of hydroxyapatite (JCPDS-PDF 74-0565). Upon increasing the soaking time, more diffraction peaks due to hydroxyapatite become evident for **Ga_1**, suggesting the crystallization of the phase. After 7 days of soaking, the (211) reflection of hydroxyapatite appeared only for **Ga_2**, while for **Ga_3** the PXRD spectrum showed only a broad band centred at 22–23° in 2*θ* characteristic of amorphous silica. Hence, the PXRD results confirm the trend observed in FTIR analysis.

**Figure 6:**
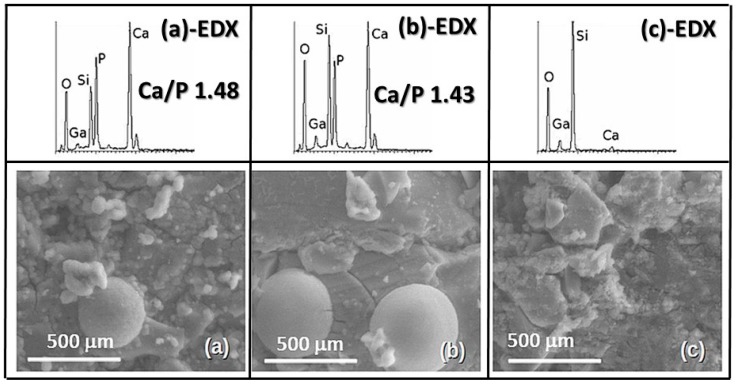
SEM micrographs and EDX analyses of (**a**) **Ga_1**, (**b**) **Ga_2**, and (**c**) **Ga_3** after 7 days of soaking in SBF.

Figure 6 shows the scanning electron microscopy (SEM) micrographs and EDX analyses of pellets of **Ga_1**, **Ga_2**, and **Ga_3** after 7 days of soaking in SBF. The **Ga_1** surface (Figure 6a) was covered by a thick layer of flake-like particles rich in Ca and P, with a Ca/P ratio of 1.48. A similar result could be observed for **Ga_2** (Figure 6b), but the glass surface seemed less homogeneous and the Ca/P ratio is 1.43. The morphology of the surface and the Ca/P ratio of **Ga_3** are instead significantly different from the previous samples (Figure 6c). These results agree with those observed by FTIR and XRD.

### 2.3. Ion Release Tests

**Figure 7:**
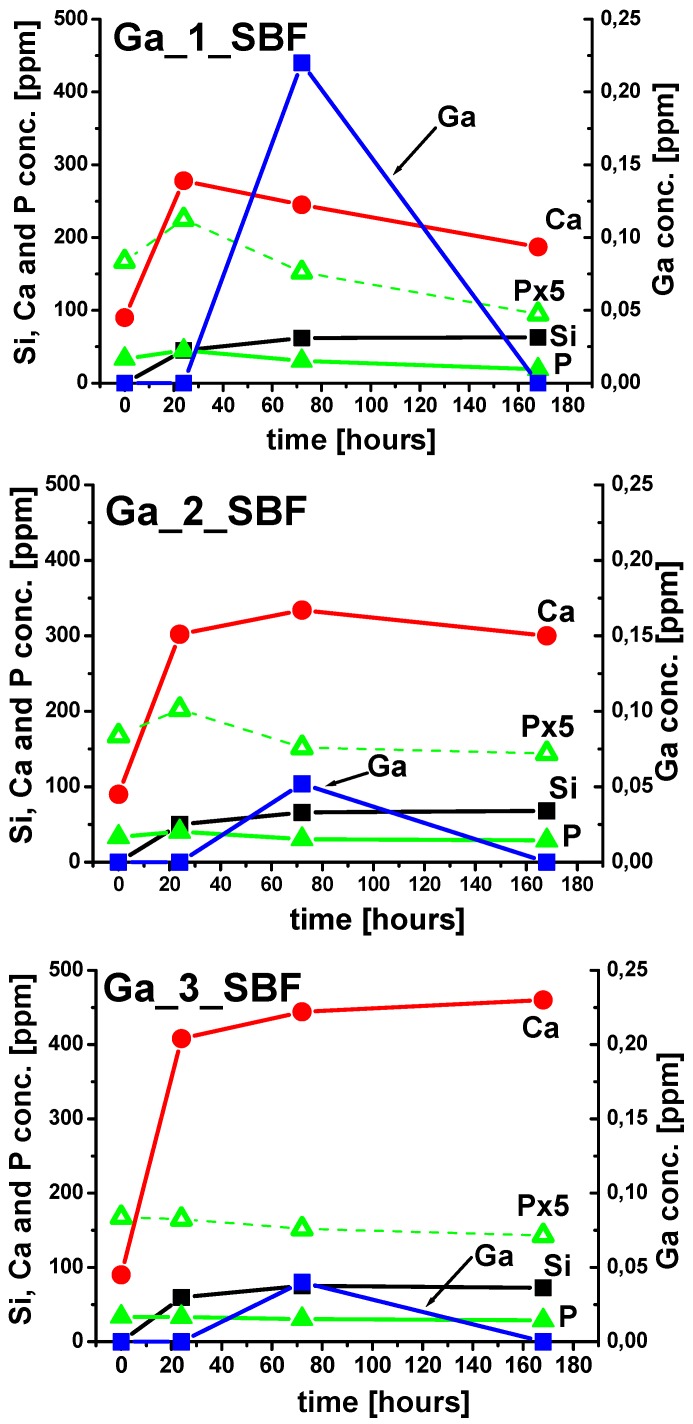
Evolution of calcium, phosphorus, silicon, and gallium concentrations with time (P × 5 represents phosphorus content magnified by a factor five for better viewing) after soaking of **Ga_1**, **Ga_2**, and **Ga_3** in SBF medium.

Changes in the SBF concentration during in vitro bioactivity tests can be used as an indirect method for understanding the process occurring on the glass surface. In fact, bioactive glasses are partially soluble; as a general requisite for bioactivity, an initial increase in Ca, P, and Si ions in solution must take place. After this, Ca and P concentrations in solution decrease, suggesting the formation of a calcium phosphate layer. The above events could be observed (see Figure 7) for **Ga_1** and **Ga_2**, while for **Ga_3** the concentration of Ca^2+^ ions increased up to 7 days, and the phosphorus concentration slightly decreased. Moreover, it is interesting to note that the maximum release of Ga^3+^ was observed after 72 h for **Ga_1**, while at longer times the concentration decreased, suggesting a partial precipitation of gallium-containing compounds.

**Figure 8:**
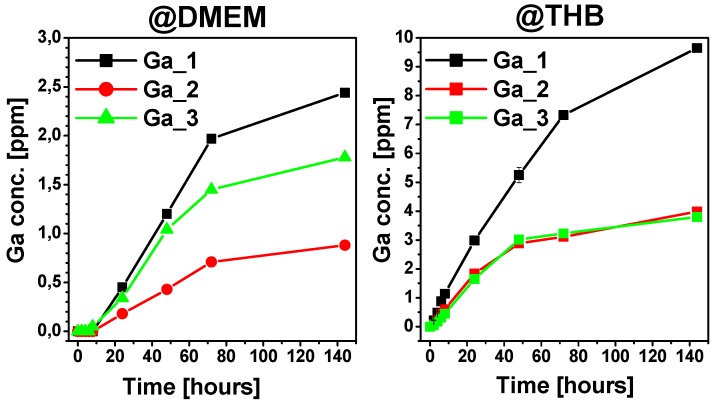
Evolution of Ga^3+^ ions release for **Ga_1**, **Ga_2**, and **Ga_3** after soaking in Dulbecco’s Modified Eagle Medium (DMEM) and Todd Hewitt Broth (THB).

To verify the simultaneous cyto-compatibility and antibacterial capacity, the cumulative release of Ga was determined, and it is reported in Figure 8. Sample **Ga_1** showed the maximum release of Ga^3+^ ions both in DMEM and THB, with the highest concentration in DMEM of about 2.5 ppm.

## 3. Discussion

The material characterization (PXRD, TEM, and N_2_ adsorption) indicated that **B** (without Ga_2_O_3_) presented a 2D hexagonal meso-structure (plane group p6mm), while the introduction of Ga_2_O_3_ decreased this ordered phase. As reported in the previous study [17], the addition of Ga_2_O_3_ up to 3.5 mol % caused the decrement of mesoporous order and of *S*_BET_, *D*_P_, and *V*_P_ with respect to the mesoporous glass without Ga_2_O_3_, while the increment of Ga_2_O_3_ from 3.5 to 5 mol % of the present study seemed not to affect the textural properties, which remained good enough to be used in bone tissue engineering.

The MAS NMR analyses performed on the samples helped us to obtain information of the medium-range glass structure. On the basis of previous studies, Ga^3+^ ions are classified as an intermediate glass network, but they can also act as network former and modifier as a function of glass composition [33,34]. The ions classified in this way often act as network formers in glasses with a high percentage of alkali and alkali-earth oxides in the composition, while they act as network modifiers in glasses with a high percentage of SiO_2_.

In our gallium-containing glass systems, the Ga^3+^ ions could not be classified as only former ions, as highlighted by comparing our single-pulse ^29^Si MAS NMR analysis and the NC values computed using Si and Ga as former ions [25]. The effect of Ga_2_O_3_ on the ^29^Si single-pulse MAS NMR data reported in Table 2 can be explained considering that when Ga^3+^ was included in the highly polymerized silica glassy matrix, these ions actuated the network modification with increased intensity of Q^3^ signal. In fact, the signal attributed to Q^3^ units shifted from −101.6 ppm for **B** to around −102.6 ppm for gallium-containing glasses, suggesting that both Ca^2+^ and Ga^3+^ ions interacted with the NBOs of the units. In particular, their formation was maximum for **Ga_3**, and this is probably due to the absence of P_2_O_5_ in the glass composition. In ^31^P MAS NMR spectra, signals due to orthophosphate Q^0^ units appeared, and these units need positive ions (i.e., Ca^2+^ and Ga^3+^) to compensate their negative charge, so in **Ga_1** and **Ga_2**, Ga^3+^ ions also act as charge compensators of PO_4_^3−^ units, causing a lower amount of gallium in the silica matrix.

Regarding Q^2^, this signal decreased in **Ga_1** and **Ga_2** with respect to **B**, which is indicative that Ga^3+^ also behaved as a network former in the structure of these samples, favouring the conversion of Q^2^ into Q^3^ species. In fact, the intermediate role of Ga^3+^ ions is well known: they play a network modifier role in the zone with a high degree of polymerization, while they act as network former in the zone with a high concentration of classical modifier (e.g., Ca^2+^ ions), with a consequent low degree of polymerization. This finding is in agreement with the increment of polymerization degree (<Q^n^>) found by classical molecular dynamic simulations performed on bioactive glasses based on the 45S5 Bioglass^®^ composition modified by the addition of 1 mol % Ga_2_O_3_. In fact, this glass system presents a high percentage of classical modifiers (Ca^2+^ and Na^+^ ions), and the Ga^3+^ ions play the role of former ions causing an enhancement of <Q^n^> value [35].

The connectivity determined by cross-polarized MAS NMR spectra was lower than in single-pulse experiments, because the first technique is mainly sensible to the glass surface rich in H nuclei [26]. In particular, as previously observed [27], it is possible to distinguish two types of Q^2^ units (Table 2): (i) Q^2^_H_ with the signal between −92 and −93 ppm, and (ii) Q^2^_Ca_ with the signal between −84 and −86 ppm.

**Figure 9:**
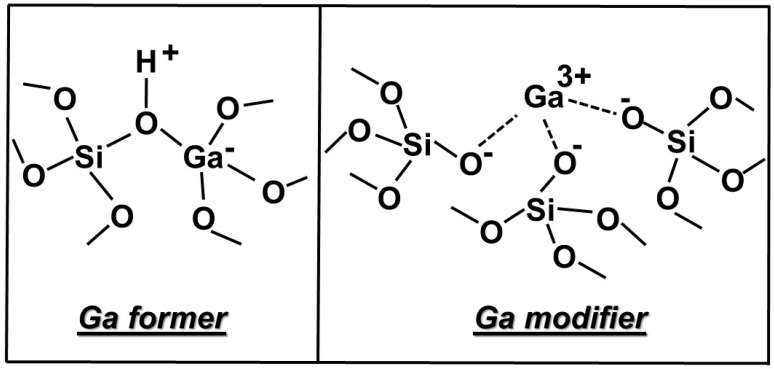
Ga^3+^ ion acting (**left**) as network former and (**right**) as network modifier on a mesoporous bioactive glass (MBG) surface network.

The shifted Q^4^ signals in cross-polarized spectra slightly increased in all cases (less negative) with respect to **B** (i.e., when gallium was included), because this ion behaves as a weak network former [36]. The role of weak network former attributed to Ga^3+^ ions could be explained by the formation of units signed as Ga former on the glass surface, as shown in Figure 9. This finding is in full agreement with a previous study on similar sol–gel glasses [34], where the Ga^3+^ ions on the surface can act as both network former and modifier (Figure 9) [37]. In particular, the picture reported on the right side of Figure 9 shows coordinatively unsaturated Ga^3+^ ions acting as modifier ions on the glassy surface; these species present on the material surface are known as cus (cation unsaturated species) [38].

The percentage of Ga acting as network former is a minority, while most of the metal ions behave as network modifiers. In fact, from the data in Table 2, it is possible to see that the percentage of Q^2^ species grew significantly for gallium-containing glass with respect to **B**. Therefore, we can conclude that Ga^3+^ ions in the glass surface mainly acted as network modifier ions. It is interesting to note that the maximum increment of Q^2^ species was detected for **Ga_1** (22.5%), which suggests that the higher amount of Ga^3+^ ions in this glass behaved as network modifiers with respect to the other gallium-containing glasses.

Phosphorous is mainly present as orthophosphate units; however, it is interesting to note that the introduction of gallium caused a slight increase in the percentage of Q^1^ units. However, they could be given by the formation of P–O–Ga, in accordance with the chemical shift determined by Ren and Eckert [39]. This trend is in line with the intermediate role of gallium: in fact, Linati et al. [40] found a similar trend studying the effect of intermediate zinc ions in bioactive glasses, where a displacement towards lower chemical shifts in ^31^P MAS NMR was detected by the increase of the percentage of ZnO in the glass [22]. The formation of P–O–Ga bridges was also detected in medium-range analysis performed using classical molecular dynamic simulations performed on bioactive glasses based on the 45S5 Bioglass^®^ composition modified by the addition of Ga_2_O_3_ [35].

The network connectivity derived from MAS NMR analysis can be used to explain the different behaviour of the samples toward the bioactivity determined by FTIR and PXRD analyses. In the case of **Ga_1** and **Ga_2**, the network connectivity was similar in both single-pulse and cross-polarized ^29^Si MAS NMR spectra, but the Q^1^ signal as determined by ^31^P MAS NMR was smaller in **Ga_2** despite a higher P_2_O_5_ % in **Ga_1**. This would indicate the presence of Ca- and P-clustered zones that are reported to favour the conversion of amorphous calcium phosphate into hydroxyapatite [20].

**Ga_3** showed lower network connectivity, but conversely it did not exhibit HCA formation: this is probably due to its phosphorous-free composition. In fact, the behaviour of glasses seems strictly related to the amount of P_2_O_5_ present in the glass composition—in particular, the absence of P_2_O_5_ in **Ga_3** avoids the presence of Ca- and P-clustered zones.

The release of Ca and Ga seems to be related to the P_2_O_5_ content. The glass with lower P_2_O_5_ mol % released Ca^2+^ ions faster, and this can be explained by the formation of Ca–P rich zones insoluble in aqueous media. The effect of P_2_O_5_ mol % in the glass composition on Ga release after 3 days is opposite to that of Ca release: in fact, **Ga_1** showed the highest P2O5 content. The interpretation of this behaviour is more complicated, and this cannot be explained only by taking into account the phosphate content.

In fact, as reported in the previous cross-polarized ^29^Si MAS NMR section, in **Ga_1** the Ga^3+^ ions on the surface acted mainly as network modifier, bringing a maximum amount of Q^2^_Ca_ species (22.5%). These species possess the lowest polymerization degree on the surface, and this explains the highest Ga release. Moreover, in order to explain the highest Ga^3+^ ions released by **Ga_1**, we can suppose a higher concentration of Ga on the glass surface with respect to the bulk with a low polymerization degree, and this enrichment was previously confirmed by X-ray photoelectron spectroscopy (XPS) analysis [34].

It is very interesting to consider that in a previous paper we reported that in ternary CaO–P_2_O_5_–SiO_2_ sol–gel glasses, the inclusion of 10% of P_2_O_5_ avoided the in vitro bioactive response in SBF [21]. Indeed, to the best of our knowledge, no MGBs containing a percentage of P_2_O_5_ as high as 10%—together with a 5% of Ga_2_O_3_ in its composition—were reported until now. Thus, the high in vitro bioactive response exhibited by **Ga_1** is surprising, as it contains just 10% of P_2_O_5_. In the mentioned paper [21], we demonstrated that a relatively smaller amount of P_2_O_5_—close to 5%---was beneficial for the glasses’ bioactivity. The reason is that P_2_O_5_ binds to calcium, forming calcium phosphate nuclei, which favour the formation of an HCA layer in SBF—characteristic of materials exhibiting in vitro bioactivity [41].

Indeed, the smaller amounts of P_2_O_5_ allow the presence of enough Ca^2+^ ions in the glass network able to be released in the surrounding medium. This process increases the super-saturation with respect to the apatite, and simultaneously provokes the formation of new surface silanol groups (Si–OH), and both factors favour HCA deposition. On the other hand, the presence of higher amounts of P_2_O_5_ binds all the Ca^2+^ ions of the glass avoiding its release, with the subsequent elimination of the bioactive response of the glass.

Nevertheless, **Ga_1** exhibited another non-favourable characteristic for bioactivity (i.e., the presence of Ga^3+^ ions). Indeed, we reported that the addition of up to 3.5% of Ga_2_O_3_ in mesoporous glasses decreased the kinetics of the bioactive response [20]. However, in this sample, the simultaneous inclusion of high amounts of both elements (P and Ga) instead produced a remarkable increase in the in vitro bioactivity. This could be explained considering that Ga^3+^ ions can bind to phosphate groups, making the release of Ca^2+^ ions into the solution easier. In the present study, we could find a glass composition (**Ga_1**) able to release a higher amount of Ga^3+^ ions in solution with respect to previous Ga-MBG samples with a maximum amounts of 3.5% of Ga_2_O_3_ (e.g., 0.22 ppm vs. <0.05 ppm) with a faster in vitro bioactivity response if compared with previous Ga-MBG samples [17]. 

The cumulative gallium release in SBF was lower than reported for the toxicity limit in blood plasma (14 ppm) [11], while in THB it was around 9.8 ppm (≈140 µM)—140 times higher than IC90 of *Pseudomonas aeruginosa* and only half that of the IC90 of *S. aureus* [42]. In view of these results, the most interesting MBG investigated for bone regeneration is **Ga_1**. In fact, this mesoporous glass presented the fastest in vitro bioactive response and potential antibacterial activity.

## 4. Materials and Methods

### 4.1. MBG Preparation

MBGs **B**, **Ga_1**, **Ga_2**, and **Ga_3** were obtained by EISA process using Pluronic^®^ P123 surfactant (P123, Sigma Aldrich, Saint Louis, MO, USA). Tetraethylorthosilicate (TEOS), triethylphosphate (TEP), calcium nitrate tetrahydrate, and gallium nitrate monohydrate (Sigma Aldrich) were used as source of SiO_2_, CaO, P_2_O_5_, and Ga_2_O_3_, respectively. These reactants were added in the desired ratio to a solution of P123 (4.5 g) and HNO_3_ 0.5 N (1.12 mL) in ethanol (85 mL). The sol was cast in Petri dish followed by gelation, ageing, and drying at room temperature for 7 days. The dried gel was calcined at 700 °C for 3 h to remove surfactant and then subjected to milling and sieving to obtain grains under 32 µM for the pellets preparation.

### 4.2. MBG Characterization

PXRD experiments were performed with a Philips X’Pert diffractometer equipped with Cu-Kα radiation (wavelength 1.5418 Å) (Eindhoven, The Netherlands). PXRD patterns were collected in the 2*θ* range between 0.6° and 8° with a step size of 0.02° and counting time of 5 s per step.

EDX analysis was performed with a JEOL 6400 microscope (Tokyo, Japan) to study the amount of silicon, calcium, phosphorus, and gallium and determine the experimental glass composition (Table 1).

Nitrogen adsorption–desorption at 77.35 K was used to determine the textural properties using a Micromeritics ASAP 2020 porosimeter (Norcross, GA, USA). Before adsorption measurement, the samples were degassed under vacuum for 24 h at 120 °C. The surface area was obtained by applying the Brunauer–Emmett–Teller (BET) method [43]. The pore size distribution was determined by the Barret–Joyner–Halenda (BJH) method [44] from the desorption branch of the isotherm. 

TEM and electron diffraction were carried out in JEOL 2000FX microscope operating at 200 kV (Tokyo, Japan).

Solid-state single-pulse MAS NMR spectra were recorded on a Bruker AV-400-WB spectrometer (Karlsruhe, Germany). Samples were spun at 10 kHz for ^29^Si and 6 kHz in the case of ^31^P. Spectrometer frequencies were set to 79.49 and 161.97 MHz for ^29^Si and ^31^P, respectively. Chemical shift values were referenced to tetramethylsilane (TMS) and H_3_PO_4_ for ^29^Si and ^31^P, respectively. The spectra were obtained also using a proton enhanced cross-polarized method, using a contact time of 1 ms. The time period between successive accumulations was 5 s and 4 s for ^29^Si and ^31^P, respectively, and the number of scans was 10,000 for all spectra.

### 4.3. In Vitro Bioactivity Tests

After calcination, glasses were ground and sieved to obtain grains with diameter lower than 32 μm. The bioactivity tests were performed by putting 50 mg of each sample in a polyethylene (PE) bottle and soaking in 10 mL of SBF [29]. The SBF solution was previously filtered with a 0.22 μm Millipore system to avoid bacterial contamination. Each sample was soaked for three different time intervals (1, 3, and 7 days) in SBF at 37 °C under continuous orbital stirring (120 rpm). After soaking, the solutions were taken out by aspiration and filtered, while the powders were gently rinsed first in distilled water and then in ethanol. Later, they were dried in a laminar airflow for 24 h. The chemical composition of the remaining solutions was analysed using an ICP spectrometer (ICP Optima 4200DV, Perkin Elmer, Waltham, MA, USA) to evaluate the changes in the concentration of calcium, silicon, phosphorus, and gallium during the in vitro bioactivity tests.

Characterization of the powders surface after in vitro bioactivity test was performed by FTIR analysis with a Nicolet Magna IR 550 spectrometer using attenuated total reflectance (ATR) setup (GMI, Ramsey, MN, USA). SEM analysis was also carried out on a JEOL 6400 microscope and a JEOL 6335F to study the morphology of the surface of the samples. EDX analysis was performed with a JEOL 6400 microscope to evaluate the amount of silicon, calcium, phosphorus, and gallium after in vitro analysis.

PXRD patterns in the 2*θ* range between 5 and 55° with a step size of 0.02° and counting time of 5 s per step were collected on the sample surface after SBF soaking to determine the crystal phase formed.

We also performed an SBF test using 50 mg of powders of each sample compacted into pellets 6 mm in diameter and 2 mm in thickness. These pellets were suspended in 10 mL of SBF, and after 7 days the surfaces were analysed with SEM to study the morphology of the samples and with EDX experiments to determine the compositional variations and the Ca/P ratio.

### 4.4. Ion Release Tests

In order to simulate the bacteria and cellular tests, as reported in Ref. [22], 50 mg of powders of each sample were compacted into pellets 6 mm in diameter and 2 mm in thickness. The tests were carried out by putting the pellet in a glass container and soaking in 2 mL of DMEM or THB and then placing in a 37 °C incubator under continuous orbital stirring (120 rpm). At various time points (2, 4, 6, 8 h and 1, 2, 3, and 7 days), the pellets were taken out of their respective containers. The solutions were analysed using ICP spectrometry (ICP Optima 4200DV) to evaluate the changes in the concentration of gallium, silicon, calcium, and phosphorus. All the pellets were placed into 2 mL of a fresh DMEM or THB solution, and this operation was performed until the last time point (7 days). This procedure allowed determination of the cumulative ion release curves shown and discussed in the Results section.

## 5. Conclusions

Three MBGs containing 5% of Ga_2_O_3_ were investigated and compared with an analogous gallium-free glass used as reference. They exhibited mesoporous structure and high *S*_BET_ surface areas (274–372 m^2^ g^−1^). NMR analysis allowed us to determine the location of Ga^3+^ ions in the glass network and to relate this position with the in vitro bioactivity and the proportion of Ga^3+^ ions released after soaking in physiological solutions. **Ga_1** was coated by an apatite-like layer after only 1 day in SBF, being the first time that an MBG containing a percentage of P_2_O_5_ as high as 10% and of Ga_2_O_3_ as high as 5% showed such a high bioactive response. This behaviour is attributed to the higher amount of modifier ions (Ca^2+^ and Ga^3+^) and consequently a more depolymerized network. On the other hand, **Ga_2** required 3 days and **Ga_3** was not coated by HCA even after 7 days of soaking. In addition, **Ga_1** was able to release relatively high amounts of Ga^3+^ ions when in contact with in vitro solutions (DMEM and THB). The Ga concentration released from **Ga_1** was inside the non-cytotoxic level reported in the literature, and was in the range of efficacy against *P. aeruginosa* bacteria and not far from the effective range reported against *S. aureus.* Thus, **Ga_1** is the first Ga-substituted MBG able to exhibit both rapid in vitro bioactivity and potential antibacterial properties with non-cytotoxic effect.

## Figures and Tables

**Table 1:** Theoretical (and experimental as obtained by energy-dispersive X-ray (EDX) analysis) molar percentage (mol %) of the different oxides in prepared mesoporous glasses.

Glasses	SiO_2_	CaO	P_2_O_5_	Ga_2_O_3_
**B**	80.0 (82.3)	15.0 (11.6)	5.0 (6.1)	0.0 (0.0)
**Ga_1**	70.0 (74.0)	15.0 (13.0)	10.0 (7.9)	5.0 (5.1)
**Ga_2**	80.0 (81.8)	12.0 (10.0)	3.0 (3.2)	5.0 (5.1)
**Ga_3**	80.0 (80.8)	15.0 (14.2)	0.0 (0.0)	5.0 (5.0)

**Table 2:** Chemical shifts δ (ppm) and relative peak areas (%) obtained by solid-state single-pulse and cross-polarized ^29^Si magic angle spinning (MAS) nuclear magnetic resonance (NMR). The areas for Q^2^, Q^3^, and Q^4^ were calculated by Gaussian line-shape deconvolutions, and their relative populations are expressed as percentages; <Q^n^> represents the silica network connectivity.

**^29^Si Single-Pulse**									
	**Q^4^**		**Q^3^**		**Q^2^**		**<Q^n^>**		
	**δ**	**Area**	**δ**	**Area**	**δ**	**Area**			
**B**	−111.5	63.4	−101.6	25.0	−91.5	11.6	3.52		
**Ga_1**	−112.1	45.9	−102.6	48.4	−89.2	5.7	3.40		
**Ga_2**	−112.0	44.0	−102.0	51.3	−89.0	4.7	3.39		
**Ga_3**	−112.0	33.2	−102.9	54.8	−89.3	11.9	3.21		
**^29^Si Cross-Polarization**									
	**Q^4^**		**Q^3^**		**Q^2^H**		**Q^2^Ca**		**<Q^n^>**
	**δ**	**Area**	**δ**	**Area**	**δ**	**Area**	**δ**	**Area**	
**B**	−110.4	18.3	−101.2	56.5	−92.4	15.8	−86.3	9.4	2.93
**Ga_1**	−107.7	20.4	−100.8	37.9	−92.9	19.0	−86.8	22.5	2.78
**Ga_2**	−106.1	20.2	−101.2	40.6	−92.3	17.6	−84.6	17.6	2.73
**Ga_3**	−106.8	22.3	−100.9	36.0	−92.7	18.1	−84.0	18.1	2.70

**Table 3:** Chemical shifts δ (ppm), relative peak areas (%), and phosphorus connectivity, <Q^n^>, obtained by solid-state single-pulse ^31^P MAS NMR.

^31^P Single Pulse					
	Q^0^		Q^1^		<Q^n^>
	δ	Area	δ	Area	
**B**	2.3	94.5	−5.7	5.5	0.06
**Ga_1**	2.0	93.3	−5.5	6.7	0.07
**Ga_2**	1.2	90.6	−5.1	9.4	0.09
